# Performance Evolution of High-Slump Concrete Under Vibration: Influence of Vibration Timing on Mechanical, Durability, and Interfacial Properties

**DOI:** 10.3390/ma18235389

**Published:** 2025-11-29

**Authors:** Shiwei Sun, Junmin Shen, Haoqin Guo, Xinxin Zheng, Rui He

**Affiliations:** 1 School of Materials Science and Engineering, Chang’an University, Middle Section of South Second Ring Road, Xi’an 710064, China; sswchd2024@163.com (S.S.); zxxchd2021@163.com (X.Z.); 2 Shaanxi School-Enterprise-Joint Research Center for Advanced Transportation Infrastructure Materials, Chang’an University, Xi’an 710064, China; 3 Shanxi Transportation Research Institute Group Co., Ltd., Taiyuan 030006, China; sjmsxjk@163.com

**Keywords:** vibration conditions, expansion joint, early-age vibration, high slump, concrete, mechanical properties, durability

## Abstract

High-slump concrete is highly sensitive to vibration due to its low viscosity and weak cohesion, factors that critically influence its performance development and long-term durability. In practice, vehicle–bridge coupled vibrations during half-width bridge construction represent a typical condition that intensifies these effects. This study investigates performance deterioration of high-slump concrete subjected to simulated vibration modes reflecting construction scenarios. Mechanical and durability properties were evaluated, and microstructural changes were analyzed using SEM. Results show that early vibration enhances compressive strength at early ages, but this benefit diminishes with curing. The bonding performance at the new–old concrete interface is highly sensitive to vibration timing, casting-to-final setting vibration greatly reduces bond strength, while initial-to-final setting vibration causes minor damage or slight improvement. Vibration modes also differently affect durability: initial-to-final setting weakens frost and abrasion resistance, whereas casting-to-final setting enhances pore structure and chloride resistance. SEM analysis reveals vibration-induced dispersion of hydration products, reduced C-S-H gel formation, and increased microcracks at the fresh–old interface. Both vibration modes further promote microcracks and porosity after freeze–thaw cycles, damaging the gel structure. Overall, this study clarifies the mechanisms by which vibration timing governs the performance evolution of high-slump concrete and provides a scientific basis for optimizing vibration procedures to ensure durability and interfacial reliability in engineering applications.

## 1. Introduction

Concrete is one of the most widely used construction materials in modern infrastructure development [1,2,3]. The early curing environment significantly affects the microstructure formation and durability of concrete, factors which are closely related to the long-term safety and reliability of transportation infrastructure [4,5,6,7]. With ongoing development of transportation facilities, maintenance has become a critical issue in the field of transportation engineering. In bridge maintenance, the method of half-width open construction is often used to reduce traffic disruption [8]. However, this method exposes freshly placed concrete to vehicle–bridge coupled vibrations during casting and curing, which can disturb the early curing environment and negatively impact the structural integrity and durability of the concrete [9,10,11,12].

Vibration often causes segregation between coarse aggregates and mortar in fresh concrete, thereby impairing its homogeneity [13,14]. This effect is more pronounced in high-slump concrete due to its lower viscosity and weaker cohesion [15,16], which reduce its ability to maintain a uniform aggregate distribution and make it more sensitive to external vibration [17,18,19]. However, in specific construction scenarios such as expansion joint repair, high-slump concrete is commonly used to ensure full encapsulation of reinforcement and effective filling of narrow spaces. This creates a technical contradiction: although high-slump concrete improves workability, it is more susceptible to vibration-induced variations in performance. Therefore, ensuring its homogeneity and durability becomes particularly critical.

Given these challenges associated with high-slump concrete under vibration, several studies have been conducted to evaluate its performance in practical repair and construction scenarios. Zhang et al. [9] studied the tensile performance of low-slump PVA-ECC with a water–binder ratio of 0.26 and found that vehicle–bridge coupled vibration had little effect on its mechanical properties. Pan [20] investigated the effect of vibration on the sulfate resistance of repair concrete with a slump of 130–150 mm and found that vibration increased cracking and accelerated sulfate attack. Harsh et al. [21] assessed the impact of traffic-induced vibrations on repair concrete with different slump levels and found that medium-slump concrete (101.6–127 mm) was less affected, while high-slump concrete showed significant performance deterioration. Kwan et al. [22] examined fresh concrete with a slump of 75 ± 25 mm and found that although no severe cracking occurred, its bond strength was notably reduced under vibration.

Beyond the influence of slump, the timing of vibration relative to the concrete setting process plays a critical role in determining its impact on performance. Studies indicate that the effect of vibration on concrete is highly time-dependent [23]. Zhang [24] divided the concrete setting process into early stage (penetration resistance 0–3.5 MPa), middle stage (3.5–28 MPa), and late stage (over 28 MPa). In the early and middle stages, concrete viscosity is low and cannot effectively resist external vibration [25,26]. As the concrete hardens, viscosity stabilizes and vibration effects gradually diminish [27]. Therefore, the most critical time for vibration-induced damage is during the early and middle stages.

Based on the understanding of time-dependent effects, related studies have further investigated the relationship between vibration and the durability of fresh concrete. Vehicle–bridge coupled vibration accelerates paste rising and aggregate settlement in fresh concrete, leading to increased segregation and reduced structural uniformity [17]. This process increases the number of harmful pores and defects, allowing aggressive agents like chloride ions to penetrate more easily and ultimately degrading concrete durability [28,29]. Research shows that the middle stage of setting and hardening is the most vulnerable to vibration [30,31]. In bridge repair zones, high reinforcement ratios are common, making high-slump concrete a frequent choice. However, studies on its performance under vibration remain limited [32,33,34]. Therefore, systematically investigating the impact of vibration on high-slump concrete under half-width construction helps optimize construction practices and enhance concrete performance.

Based on this background, this study prepared high-slump concrete and simulated vehicle–bridge coupled vibration to investigate its effects on mechanical and durability properties. Two vibration loading modes were designed according to the initial and final setting times: one applied vibration throughout the casting to final setting process, and the other applied vibration only during the initial to final setting stage. The effects of vibration were evaluated in terms of compressive strength, bond-splitting strength, freeze–thaw resistance, chloride ion penetration resistance, and abrasion resistance. Scanning electron microscopy (SEM) was also used to analyze microstructural changes and reveal the mechanisms behind performance evolution.

## 2. Materials and Methods

### 2.1. Materials

Ordinary Portland cement (P·O 42.5) produced by the Conch Cement Plant in Xi’an, Shaanxi Province, was used in this study, and its physical properties are listed in Table 1. The fine aggregate was river sand from the Ba River in Xi’an, with a fineness modulus of 2.4. The coarse aggregate consisted of crushed limestone with particle sizes of 5–10 mm and 10–20 mm, mixed at a ratio of 3:7. The bulk density of the coarse aggregate was 2730 kg/m^3^. A polycarboxylate-based superplasticizer (SP) produced by Sika (Switzerland), with a water-reducing rate of 25%, was used as the admixture.

### 2.2. Mix Proportion

To study the effect of vibration on high-slump concrete, a concrete mix with a slump of 180 mm was designed according to the slump classification standards in the Standard for quality control of concrete (GB 50164-2011) [35]. The mix proportions are shown in Table 2.

### 2.3. Vibration Modes

**Figure 1:**
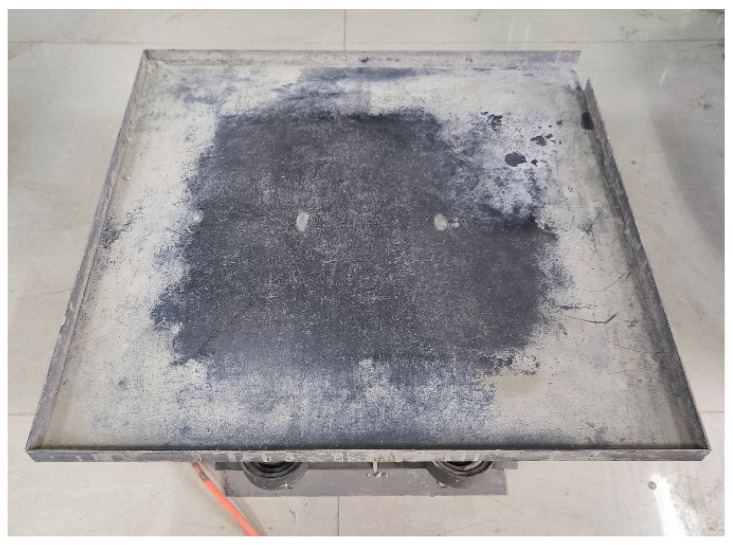
Concrete vibration table.

**Figure 2:**
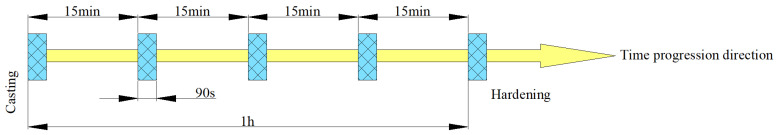
Schematic diagram of disturbance periods for fresh concrete.

After casting, the specimens were subjected to external vibration on a 1m × 1 m horizontal electric vibration table manufactured by Guangdong Dongguan Jingyu Instrumentation Equipment Technology Co., Ltd., China, as shown in Figure 1. The vibration conditions were determined with reference to the Vibrating table for concrete test (JG/T 245-2009) [36] and further adjusted according to the actual characteristics of vehicle-induced bridge vibrations under half-width traffic. Previous studies and field monitoring [23] have shown that traffic-induced bridge vibrations are mainly concentrated in the low-frequency range of 2–10 Hz. Considering actual traffic conditions, a representative frequency of 5 Hz was selected for simulation, corresponding to 18,000 cycles/h. To achieve this, a horizontal electric vibration table with a rated frequency of 50 Hz was employed, with the vibration amplitude controlled at 2 mm. The vibration was applied in an intermittent mode: four cycles per hour, each consisting of 13.5 min of rest and 90 s of vibration, ensuring approximately 18,000 vibration cycles per hour, as illustrated in Figure 2.

To simulate different disturbance scenarios at early ages of concrete, two vibration loading modes were designed. The first mode applied continuous vibration from casting to final setting (penetration resistance 0–28 MPa), representing continuous disturbance caused by immediate traffic loading after casting. The second mode applied vibration from initial to final setting (penetration resistance 3.5–28 MPa), representing delayed disturbance by traffic after the concrete had reached initial setting. A static control group was additionally established for comparative analysis. During specimen preparation for this group, consolidation was performed using a steel tamping rod in accordance with the Chinese national standard Testing Methods of Cement and Concrete for Highway Engineering (JTG 3420-2020) [37].

Penetration resistance was adopted as the division criterion because it quantitatively characterizes the hydration process, ensuring comparability between different batches. According to the Standard for test method of performance on ordinary fresh concrete (GB/T 50080-2016) [38], the setting time of the concrete was determined. The times corresponding to penetration resistances of 3.5 MPa and 28 MPa were recorded as the initial and final setting times. Based on the preliminary test results, the initial and final setting times of the concrete used in this study were 465 min and 690 min, respectively. Detailed vibration conditions for each case are summarized in Table 3.

The specimens subjected to vibration were immediately transferred to a standard curing room (20 ± 3 °C, relative humidity > 95%) and cured until the designated testing ages. The control group specimens were cured under the same conditions to ensure consistency.

**Figure 3:**
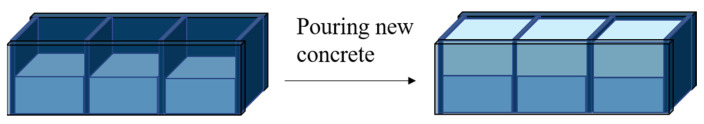
Bond-splitting specimen casting process.

Different sizes of specimens were prepared for the tests. Compressive strength tests used cubes measuring 100 mm × 100 mm × 100 mm. Bond-splitting strength tests used cubes of the same size (100 mm × 100 mm × 100 mm) composed of old and fresh concrete; the specimen preparation process is shown in Figure 3. Freeze–thaw cycle tests used prisms measuring 400 mm × 100 mm × 100 mm. Chloride ion penetration tests used disk-shaped specimens with a diameter of 100 mm and height of 50 mm. Abrasion resistance tests used cubes measuring 150 mm × 150 mm × 150 mm.

### 2.4. Mechanical Properties Test

#### 2.4.1. Compressive Strength Test

According to the JTG 3420-2020, concrete specimens with dimensions of 100 mm × 100 mm × 100 mm were prepared. The compressive strength of the concrete was tested using a TYE-2000B compression testing machine manufactured by Wuxi Jianyi Instrument & Machinery Co., Ltd., China, at a loading rate of 0.5 MPa/s until specimen failure, and the ultimate load *f* at failure was recorded. Three specimens were prepared for each group, and the measured value was taken as the arithmetic mean of the three specimens. Tests were conducted at concrete ages of 1, 7, 14, and 28 days, respectively. The calculation method for the disturbance coefficient of compressive strength is shown in Equation (1):

(1) $$K_{C}=\left(1-\frac{f^{’}}{f}\right)\times 100\%$$

where *K_c_* is the disturbance coefficient of compressive strength, %; *f’* is the strength of vibrated concrete, MPa; and *f* is the strength of static (non-vibrated) concrete, MPa.

#### 2.4.2. Bond-Splitting Strength Test

**Figure 4:**
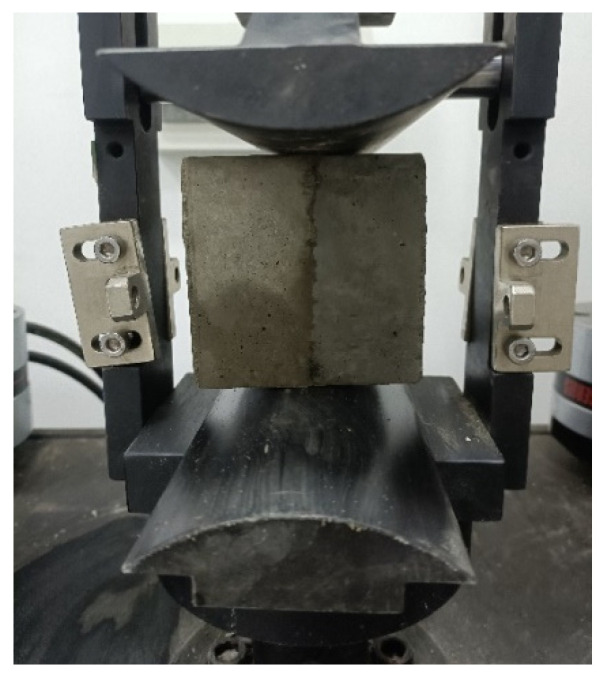
Concrete bond-splitting test process.

According to the JTG 3420-2020, the bond-splitting strength was tested using a universal testing machine with a capacity of 100 kN manufactured by Wuxi Jianyi Instrument & Machinery Co., Ltd., China. Ordinary concrete prisms with dimensions of 100 mm × 100 mm × 100 mm were prepared as old concrete and cured under standard conditions for 28 days. The old concrete specimens were then cut in half using a cutting machine to obtain two 100 mm × 100 mm × 50 mm concrete blocks. The cut surface was used directly as the casting surface, placed facing upward in the mold, and new concrete was poured on top. The specimens were demolded after 24 h, and interfacial bond strength tests were conducted using the universal testing machine at the specified curing ages. The loading rate was maintained at 0.5 MPa/s until specimen failure, and the ultimate load at failure was recorded. Three specimens were prepared for each group, and the measured value was taken as the arithmetic mean of the three specimens. The loading points of the splitting fixture were kept in contact with the bonding interface between the old and new concrete [39,40], as shown in Figure 4. Tests were conducted at concrete ages of 1, 7, 14, and 28 days, respectively. The calculation of the disturbance coefficient for bond-splitting strength (Kbs) is similar to that for compressive strength.

The double-layer concrete specimens used in this study are not standard specimens defined in JTG 3420-2020. This configuration was selected because the study focuses on the effect of vibration on interfacial bonding performance, and the double-layer design can simulate the actual interlayer interface that exists during vibration. This enables the splitting failure to concentrate at the interface, allowing a more accurate assessment of vibration-induced bonding degradation. The materials, mixing, and curing procedures still follow the general requirements of JTG 3420-2020, making this specimen form both necessary and reasonable.

### 2.5. Durability Test

#### 2.5.1. Freeze–Thaw Cycle Test

**Figure 5:**
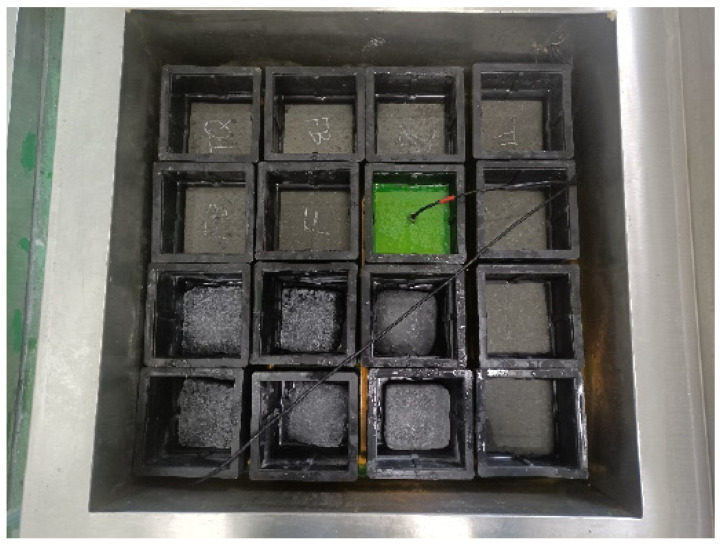
Concrete freeze–thaw cycle test.

According to the JTG 3420-2020 standard, the rapid freeze–thaw method was used to evaluate the frost resistance of concrete. Concrete specimens with dimensions of 100 mm × 100 mm × 400 mm were prepared and tested for freeze–thaw resistance after 28 days of curing, as shown in Figure 5. Each freeze–thaw cycle lasted 4 h, and the mass and dynamic elastic modulus (A dynamic slump tester manufactured by Tianjin Lewei Co., Ltd., China) of the specimens were measured every 25 cycles. The test stops if the relative dynamic elastic modulus falls below 60% or the mass loss rate exceeds 5%. Otherwise, the test continues until 200 freeze–thaw cycles are completed. Three specimens were prepared for each group, and the measured value was taken as the arithmetic mean of the three specimens. The calculation methods for mass loss rate and relative dynamic elastic modulus are shown in Equations (2) and (3):

(2) $$W_{n}=\frac{m_{0}-m_{1}}{m_{0}}\times 100\%$$

where *W_n_* is the mass loss rate of the specimen after *n* freeze–thaw cycles, %; *m_0_* is the initial mass before the freeze–thaw test, kg; and mn is the mass after *n* freeze–thaw cycles, kg.

(3) $$P=\frac{f_{n}^{2}}{f_{0}^{2}}\times 100\%$$

where *P* is the relative dynamic elastic modulus after *n* freeze–thaw cycles, %; *f_0_* is the transverse fundamental frequency before freeze–thaw testing, Hz; and *f_n_* is the transverse fundamental frequency after *n* freeze–thaw cycles, Hz.

#### 2.5.2. Chloride Ion Penetration Test

**Figure 6:**
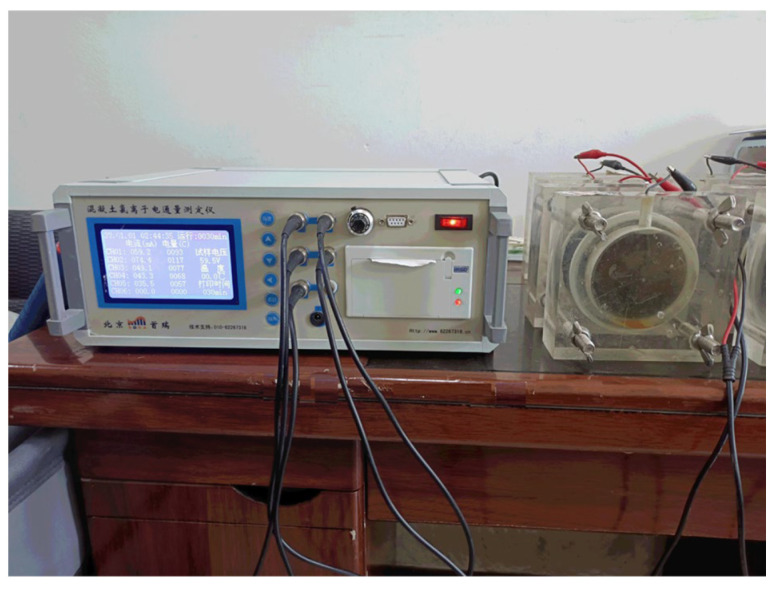
Concrete chloride penetration resistance test.

According to the JTG 3420-2020, the chloride penetration resistance of concrete was evaluated using the electric flux method, as shown in Figure 6. Disk-shaped specimens with a diameter of 100 ± 1 mm and a height of 50 ± 2 mm were prepared and cured for 28 days. After curing, the sides of each specimen were sealed with two layers of epoxy resin. A 3% sodium chloride (NaCl) solution was used to test the chloride ion resistance. The total electric charge passed through the specimen within 6 h was used as the evaluation index. The current passing through the specimen was measured every 30 min using a concrete chloride ion electric flux tester manufactured by Beijing Shourui Co., Ltd., China. The accumulated current over 6 h was recorded as the electric flux. Three specimens were prepared for each group, and the measured value was taken as the arithmetic mean of the three specimens. The calculation of electric flux is shown in Equation (4):

(4) $$Q=900\left(I_{0}+2I_{30}+2I_{60}+2I_{90}+I_{120}+\cdot \cdot \cdot \cdot \cdot \cdot +2I_{300}+2I_{330}+I_{360}\right)$$

where *Q* is the total electric flux through the specimen, C; *I_0_* is the initial current, A; *I_t_* is the current at time *t* minutes, A.

#### 2.5.3. Abrasion Resistance Test

**Figure 7:**
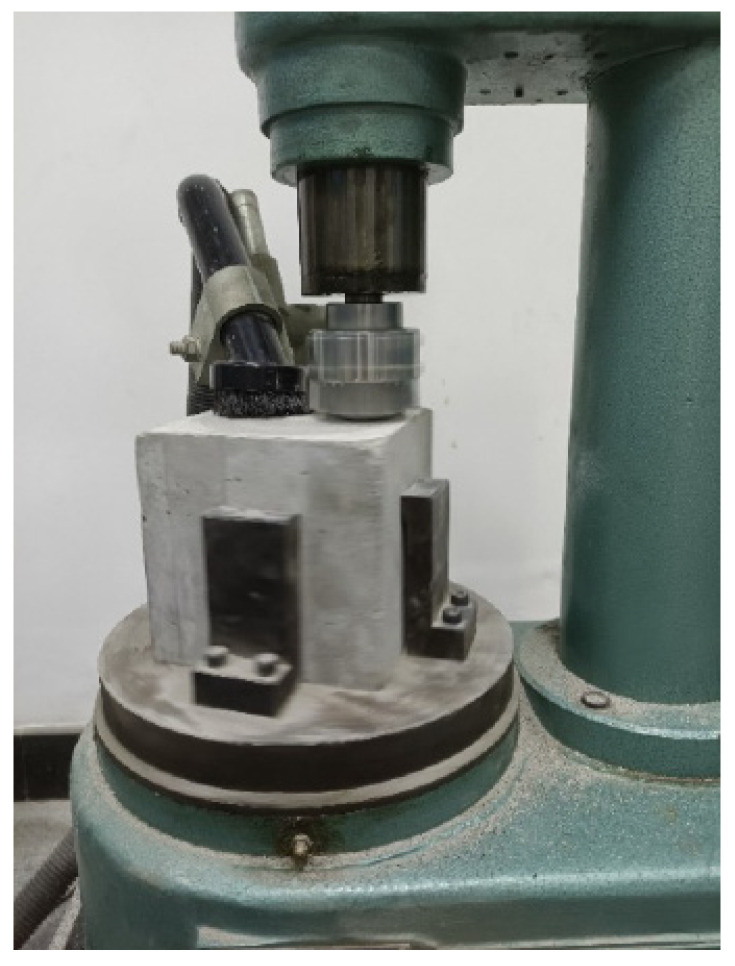
Concrete abrasion resistance test.

Vibration significantly affects the compactness of the formed surface of concrete, which in turn influences its abrasion resistance. To study the impact of vibration on the abrasion resistance of concrete, the surface of 28-day-old concrete was used as the abrasion surface of the specimen [41]. According to the JTG 3420-2020 standard, concrete specimens with dimensions of 150 mm × 150 mm × 150 mm were prepared and cured for 28 days. The abrasion resistance of the concrete was then tested using a concrete abrasion testing machine manufactured by Wuxi Jianyi Instrument & Machinery Co., Ltd., China, as shown in Figure 7. Under a load of 200 N, the specimen was ground for 30 revolutions, and the mass at this stage was recorded as *m*_1_. Then, the specimen was further ground for 60 additional revolutions under the same load, and the mass was recorded as *m_2_*. Three specimens were prepared for each group, and the measured value was taken as the arithmetic mean of the three specimens. The mass loss per unit area of concrete due to abrasion is calculated using Equation (5):

(5) $$G_{c}=\frac{m_{1}-m_{2}}{A}$$

where *G_c_* is the mass loss per unit area due to abrasion, kg/m^2^; *m_1_* is the initial mass, kg; *m_2_* is the mass after abrasion, kg; A is the surface area of the specimen, m^2^.

### 2.6. Microstructure Test

**Figure 8:**
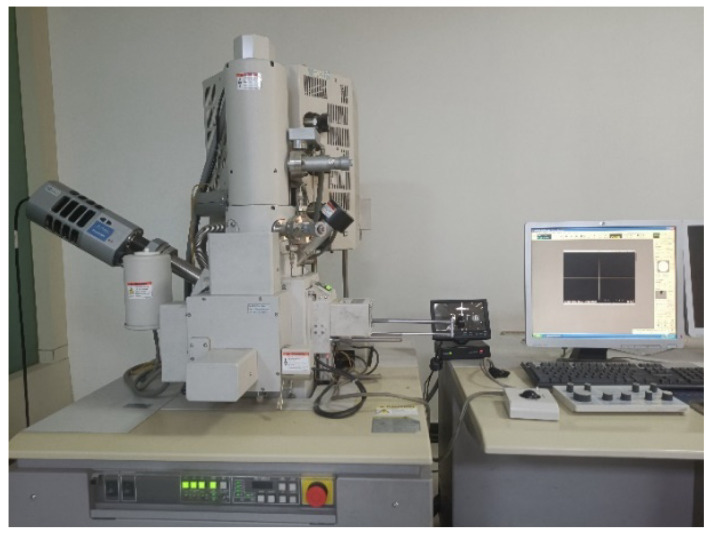
SEM test.

Due to the damage caused by freeze–thaw cycles to the internal structure of concrete, microscopic analysis was conducted on concrete specimens after the freeze–thaw test. Specifically, a cube measuring 5 mm × 5 mm × 2 mm was selected from the center of the fractured freeze–thaw specimen for further analysis. The cube was soaked in absolute ethanol until cement hydration was fully stopped. After that, the sample was dried and coated with gold. Then, a scanning electron microscope (S-4800) manufactured by Hitachi, Japan, was used to scan the surface of the specimen to observe pore structures and the morphology of hydration products, as shown in Figure 8. In addition, since the bonding performance between repair concrete and old concrete significantly affects repair quality, the interface between the new and old concrete was also analyzed using a scanning electron microscope to explore the bonding mechanism.

## 3. Results

### 3.1. Mechanical Properties

#### 3.1.1. Compressive Strength

**Figure 9:**
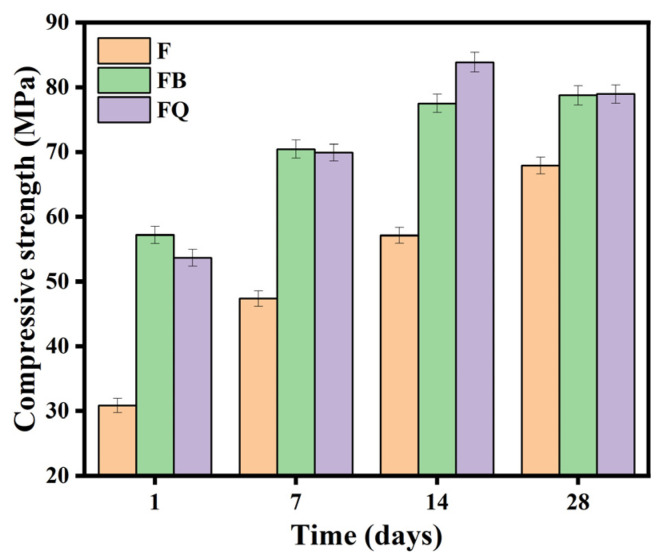
Variation diagram of compressive strength of concrete.

**Figure 10:**
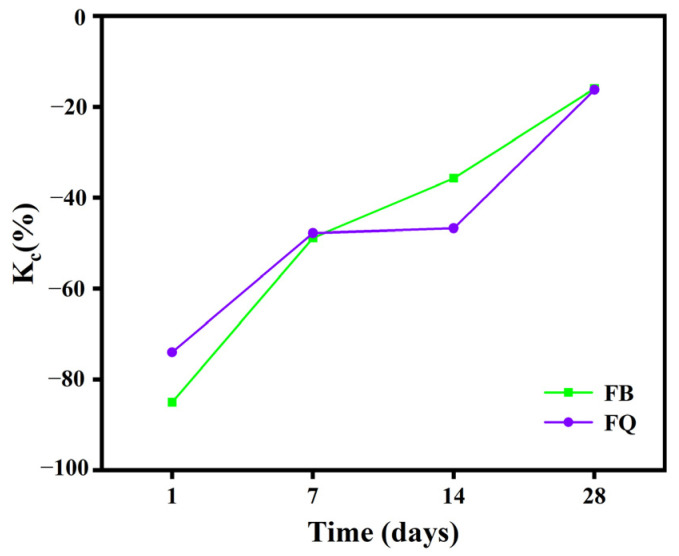
Disturbance coefficient diagram of compressive strength of concrete.

The compressive strength of concrete under different vibration methods is shown in Figure 9. Vibration during the setting and hardening period significantly increased the early compressive strength of the concrete. At 1 day age, the compressive strength of groups FQ and FB increased by 85.1% and 74.6%, respectively, compared to group F. As the curing age increased, the compressive strength of all groups improved significantly, but the differences gradually decreased. At 28 days age, the compressive strength of groups FQ and FB was 16.1% and 16.2% higher than that of group F, indicating that the effect of early vibration on compressive strength gradually weakens with time. The disturbance coefficient of compressive strength for different vibration methods is shown in Figure 10 (The smaller the absolute value of the disturbance coefficient, the smaller the strength difference between the test group and the control group). With increasing age, the disturbance coefficient shows different trends for each vibration method. At 1 day age, the disturbance coefficient is significantly negative. As the curing age increases, the disturbance coefficient gradually decreases.

**Figure 11:**
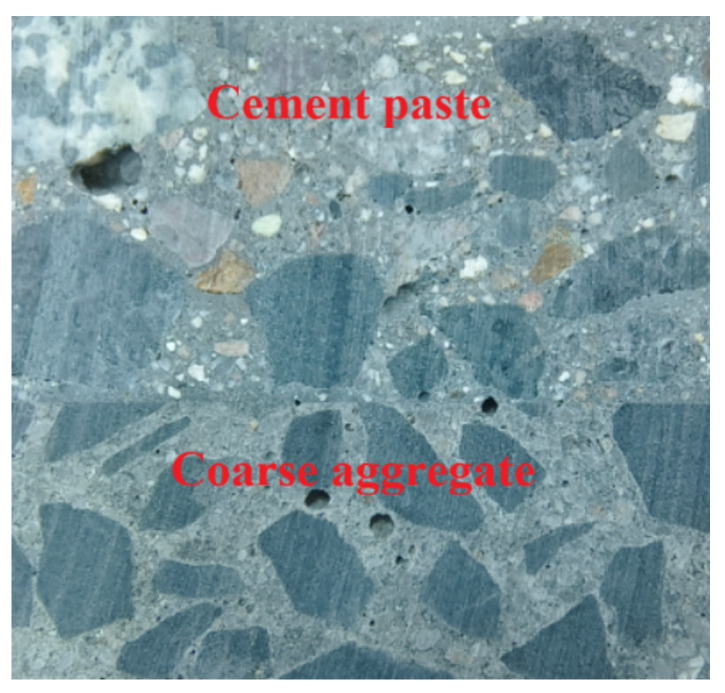
Diagram of paste–aggregate segregation.

At the early stage, the concrete matrix remains highly fluid, and continuous vibration promotes the redistribution of cement paste and fine particles around coarse aggregates, effectively filling micro-voids and expelling entrapped air, thereby enhancing the overall compactness of the concrete [15,42]. Meanwhile, due to the low cohesiveness of high-slump concrete, segregation tends to occur during vibration, causing the paste to separate from the coarse aggregates in the upper region and locally increasing the water–cement ratio (as shown in Figure 11). This condition accelerates the evaporation of surface water and effectively reduces the actual water–cement ratio of the entire system, further promoting the development of early compressive strength. Therefore, vibration-induced paste densification, microstructural rearrangement, and the reduction in the effective water–cement ratio collectively dominate the early strength enhancement, enabling the vibrated specimens to exhibit a significant strength advantage at 1 day, with the positive effects outweighing the adverse influence of initial segregation.

However, this strength enhancement is clearly time-dependent. As curing progresses, the negative effects of segregation gradually emerge. Vibration causes paste loss in the lower region, weakening the effective bonding between cement and aggregates and leading to non-uniform hydration during the early hardening stage. In addition, continuous vibration disturbs the initial formation of hydration products, thereby limiting the potential for later strength development.

#### 3.1.2. Bond-Splitting Strength

**Figure 12:**
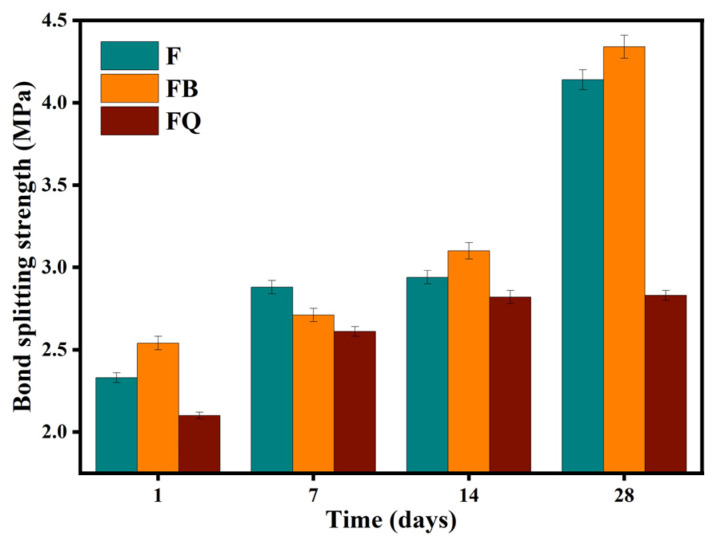
Bond-splitting strength diagram of concrete.

**Figure 13:**
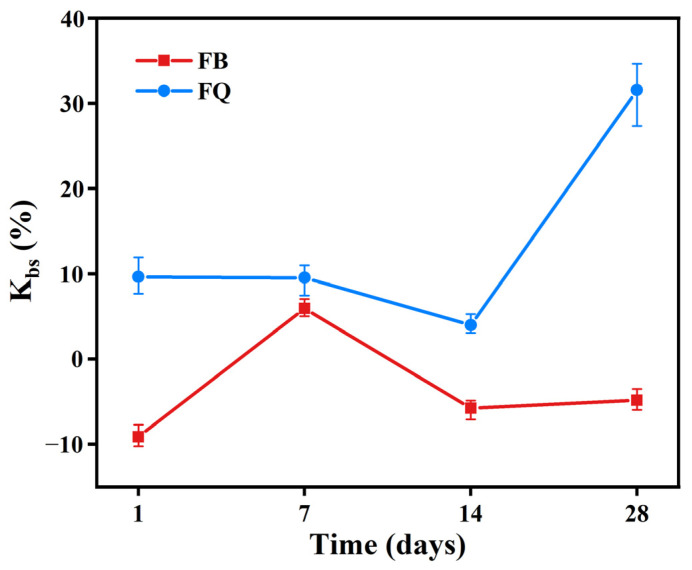
Disturbance coefficient curve of concrete bond-splitting strength.

The bond-splitting strength of the interface between new and old concrete is an important indicator for evaluating the repair and reinforcement effect of concrete structures [43]. The bond-splitting strength of concrete under different vibration methods is shown in Figure 12, and the disturbed coefficient curves of bond-splitting strength under different vibration methods are shown in Figure 13. It can be seen that, within the first 14 days of curing, the disturbed coefficients of bond-splitting strength for groups FB and FQ ranged between −10% and 10%, which are relatively small and statistically insignificant. During this curing period, the cement hydration reaction is not yet complete, and the microstructure of the cement paste is relatively loose. Although vibration improves concrete compactness, the bond strength between cement and aggregate is not fully developed, so the effect of vibration on bond-splitting strength is not significant.

At the 28-day curing age, the bond-splitting strength of group FB increased significantly, with a disturbed coefficient of −5.8%. At this time, the cement hydration reaction is nearly complete, and the microstructure of the cement paste has stabilized. Vibration effectively removes internal bubbles and voids in the concrete, enhancing the bond strength between cement and aggregate, thus improving the bond-splitting strength. In contrast, the bond-splitting strength of group FQ did not show a significant increase at 28 days, and its disturbed coefficient reached as high as 32.8%. This is because vibration before initial setting in this mode caused structural defects in the cement hydration products, limiting the later growth of bond-splitting strength. Moreover, long-duration vibration cause some paste loss or uneven cement hydration, suppressing further strength development and resulting in insufficient bond strength at the interface between new and old concrete in later stages [33].

**Figure 14:**
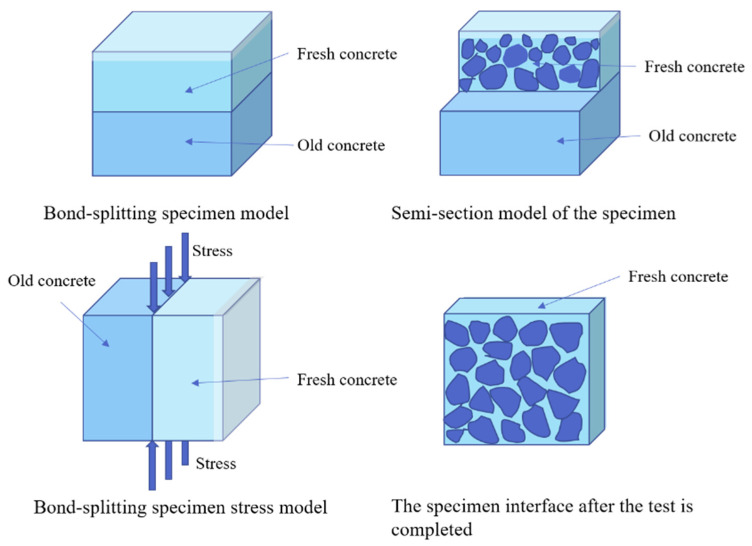
Failure mechanism model of concrete bond-splitting specimens.

The failure mechanism model of the concrete bond-splitting test specimen is shown in Figure 14. The bond-splitting specimen model demonstrates the actual bonding condition between new concrete on the upper layer and old concrete on the lower layer, reflecting real engineering situations. The half-section view of the specimen clearly shows the distribution of coarse aggregates in the new concrete, helping to understand the structural influence on bond performance. The stress model illustrates the stress applied vertically to the interface between new and old concrete during the test, simulating the actual loading condition. The post-test interface image shows the distribution of coarse aggregates in the new concrete and possible failure features.

From a microscopic perspective, without an interface bonding agent, the bond between new and old concrete mainly relies on mechanical interlocking. This interlocking is caused by the interlocking of components from both new and old concrete. Increasing the roughness of the interface enhances the mechanical interlocking effect between the new and old concrete, which helps improve the bond strength. Mechanical interlocking between irregular surfaces is the dominant bonding mechanism between new and old concrete [44].

According to SEM test results, the microstructure changes at 28 days of age were evaluated for new and old concrete specimens subjected to vibration from casting to final setting (FQ group) and for a non-vibrated control group (F). The study further explored how the vehicle–bridge coupled vibration during this period affected the bond strength at the interface between new and old concrete.

**Figure 15:**
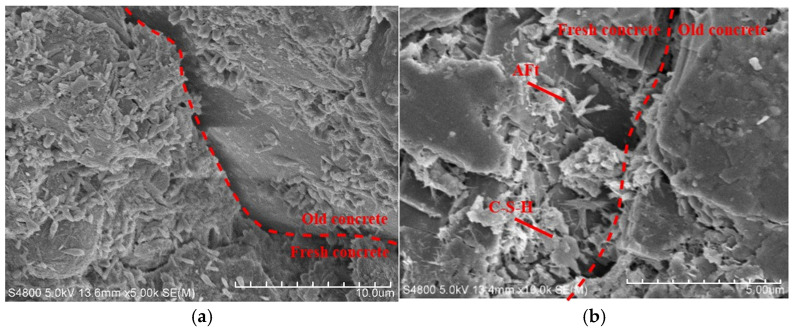
Microstructure of fresh-to-old interface of concrete with vibration from concrete casting to the final setting stage: (**a**) 5000 times SEM image; (**b**) 10,000 times SEM image. Legend: The red dashed line in the figure is used to indicate the fresh-to-old interface.

The microstructure of the fresh-to-old concrete interface under vibration from casting to final setting is shown in Figure 15. In the FQ group, hydration products at the interface were scattered, with a small amount of calcium silicate hydrate (C-S-H) gel formed and an unclear flocculent structure. This indicates weak bonding between cement and aggregates, lacking a cohesive composite structure. The amounts of plate-like calcium hydroxide (CH) and needle-like ettringite (AFt) were also reduced and dispersed independently, failing to effectively promote further bonding between cement and aggregates. Additionally, many fine cracks appeared at the interface, and these cracks and voids were not filled by hydration products, showing that the vehicle–bridge coupled vibration had a negative effect on the interface structure, leading to reduced bond strength.

**Figure 16:**
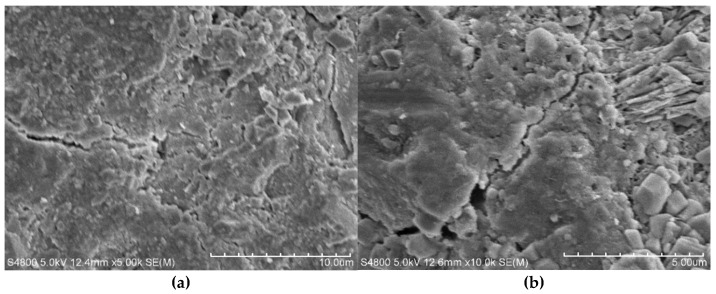
Microstructure of fresh-to-old interface of concrete without vibration: (**a**) 5000 times SEM image; (**b**) 10,000 times SEM image.

In contrast, the non-vibrated control group (F), shown in Figure 16, had a dense accumulation of flocculent C-S-H gel at the fresh-to-old interface. These hydration products effectively filled microscopic pores in the interface region and promoted bonding between cement and aggregates. The C-S-H gel was evenly distributed, wrapping other hydration products, resulting in relatively fine cracks mostly covered by hydration products. This suggests that without vehicle–bridge coupled vibration, the interface maintained a relatively complete and dense structure, helping to preserve the bond strength without significant negative impact.

These microstructural differences reveal the significant influence of vehicle–bridge coupled vibration from casting to final setting on the concrete interface. Vibration caused uneven formation of hydration products, crack development, and missing hydration products at the interface, directly weakening the bond strength. Conversely, the absence of vibration allowed the formation of a dense hydration product layer, enhancing the cement-aggregate bond and effectively maintaining bond strength.

### 3.2. Durability Properties

#### 3.2.1. Frost Resistance

**Figure 17:**
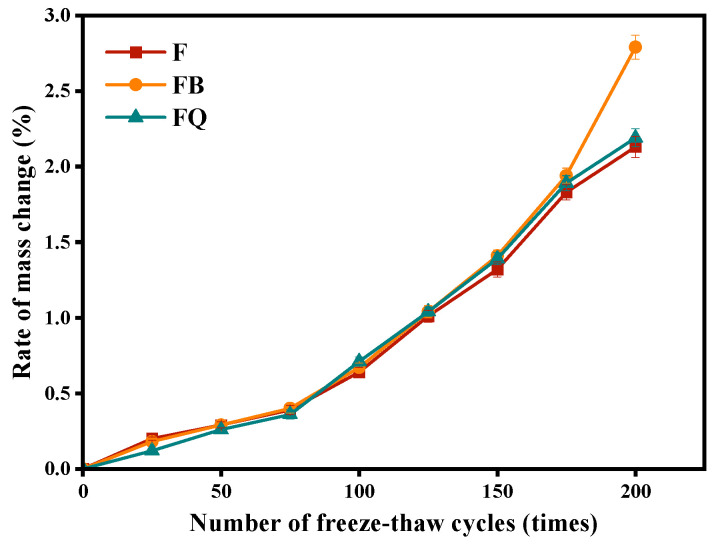
Mass loss rate of concrete after freeze–thaw cycles.

The change in mass loss rate of concrete in groups F, FB, and FQ under different freeze–thaw cycles is shown in Figure 17. Group F, without vibration, has a relatively stable initial structure. With the freeze–thaw cycles, repeated freezing and thawing of pore water causes a gradual increase in mass loss rate, reaching 2.1% after 200 cycles. Group FQ, vibrated throughout the entire process, disturbs the structural uniformity to some extent. Its mass loss trend is similar to group F, but the mass loss rate after 200 cycles is slightly higher than group F. In contrast, group FB shows no significant difference in mass loss rate from the other two groups in the early freeze–thaw cycles. However, in the later stage (cycles 175–200), its mass loss rate sharply increases, reaching 2.8% after 200 cycles. This is because vibration from initial to final setting causes more microcracks in the semi-solid concrete with poor bonding, creating weak points inside the concrete. As freeze–thaw cycles increase, these microcracks expand and connect more easily under pore water pressure, accelerating structural damage and causing much higher mass loss than groups F and FQ. This highlights the significant difference of group FB in later freeze–thaw cycles.

**Figure 18:**
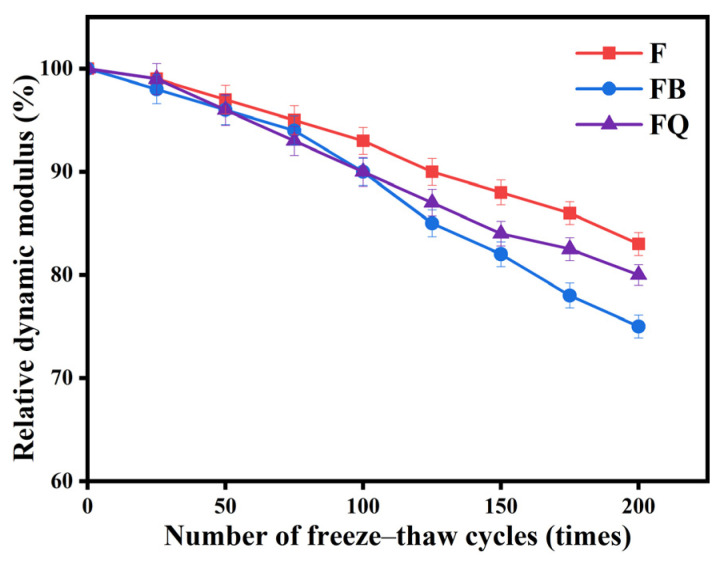
Relative dynamic elastic modulus of concrete after freeze–thaw cycles.

Figure 18 shows the relative dynamic elastic modulus of vibrated concrete after freeze–thaw cycles. With increasing freeze–thaw cycles, the dynamic elastic modulus of normal concrete changes slowly within the first 200 cycles. Different curing environments also cause significant differences in relative dynamic elastic modulus. Group FB shows the fastest decline in relative dynamic elastic modulus. This is because vibration from initial to final setting increases microcracks and pores inside the semi-solid concrete. During freeze–thaw, moisture gathers in these weak points, causing stress concentration and faster crack growth. Additionally, vibration affects the distribution of hydration products. Key bonding hydration products, such as C-S-H gel, lose continuity and density, making it difficult to resist freezing expansion stress. This shows that vibration during the initial to final setting stage has the greatest negative impact on concrete freeze–thaw resistance. Therefore, in cold regions or winter construction, bridge coupled vibration during this stage can cause more significant damage to concrete.

**Figure 19:**
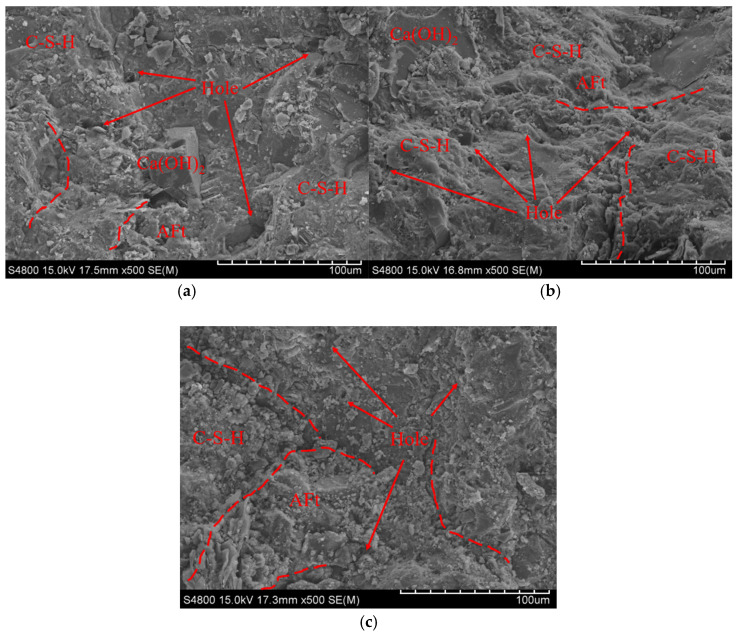
Microstructure of vibrated concrete under the combined effects of vibration and freeze–thaw cycles: (**a**) F, (**b**) FB, (**c**) FQ. Legend: The red dashed lines in the figure indicate the internal interfaces or pore regions of the concrete, and the red arrows point to the distribution of holes.

The microstructural changes in concrete under the combined effects of vibration and freeze–thaw cycles are shown in Figure 19. After freeze–thaw cycling, cracks and pores appeared inside the concrete to varying degrees, indicating damage to its internal structure. As shown in Figure 19a, the control group’s concrete surface remained relatively smooth and dense, with fewer cracks, suggesting good freeze–thaw resistance. Large calcium hydroxide (CH) crystals wrapped in calcium silicate hydrate (C-S-H) gel and some ettringite (Aft) were observed, indicating a relatively uniform distribution of hydration products and high structural integrity. Figure 19b shows the microstructure of concrete vibrated during the initial to final setting stage. After freeze–thaw action, its surface became noticeably rougher, and the number of large pores increased significantly. This suggests that vibration during this stage weakened the stability of the gel structure and caused internal loosening. Figure 19c displays the microstructure of concrete subjected to continuous vibration from casting to final setting. After freeze–thaw cycles, its internal structure appeared loose, CH crystal size was reduced, cracks were widespread on both the surface and internal areas, and pore connectivity increased, leading to a decline in overall structural integrity. These results indicate that vibration adversely affects the microstructural stability of concrete. Disturbances before the complete hardening of the cementitious matrix reduce the bonding strength of the C-S-H gel, making it less capable of resisting the expansive stresses caused by freeze–thaw cycles [45]. Consequently, more severe microstructural deterioration is observed under freeze–thaw conditions.

#### 3.2.2. Chloride Penetration Resistance

**Figure 20:**
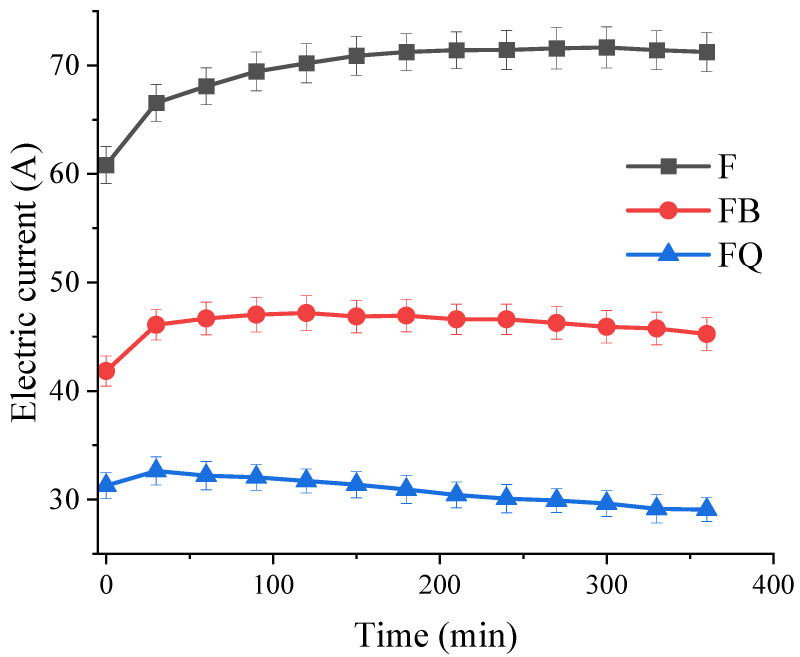
Variation pattern of internal current in concrete under different vibration methods.

As shown in Figure 20 and Table 4, the electric flux and current variation in concrete under different vibration conditions exhibit significant differences, clearly reflecting the influence of vibration timing and duration on chloride penetration resistance. For group F, the current values remained at a relatively high level throughout the test and gradually stabilized over time, with a total electric flux of 1578 C, corresponding to a low resistance grade. This indicates that its pore structure was not further modified, resulting in lower resistance to chloride transport and the poorest durability performance.

In contrast, the current values of group FB were significantly lower than those of group F at the early stage, with an electric flux of 1044 C. This suggests that vibration applied during this period promoted matrix densification and reduced pore connectivity, thereby inhibiting chloride penetration in the early phase. However, group FB exhibited deterioration in other durability tests (such as freeze–thaw and abrasion resistance). This implies that vibration during the initial setting stage tended to generate microcracks and uneven distribution of hydration products at the interfaces. These microstructural defects were not fully reflected in the electric flux results, but they weakened resistance to freeze–thaw and mechanical wear.

The most remarkable result was observed in group FQ. Its current values remained the lowest with minimal fluctuations throughout the test, with a total electric flux of only 695 C, corresponding to a very low permeability grade. This indicates that continuous vibration applied while the matrix was still in a fluid state significantly improved overall compactness and interfacial bonding, thereby reducing the probability of forming chloride transport channels and achieving the best blocking effect.

It is noteworthy that the FQ group exhibited relatively good chloride penetration resistance despite its lower splitting tensile strength and frost resistance. This is because continuous vibration was applied while the matrix was still in a highly fluid state, leading to two competing structural evolution processes occurring simultaneously: on one hand, early vibration promoted the expulsion of free water and microbubbles within the paste, and the aggregates rearranged to more favorable positions for compaction, significantly reducing the overall porosity and the number of connected pores while increasing the tortuosity of the pore network. These changes are the dominant factors affecting the electrical flux test; thus, even in the presence of local interface defects, the overall ion transport paths were lengthened and distorted, resulting in the lowest total electrical flux and relatively good chloride penetration resistance for the FQ group. On the other hand, continuous vibration induced strong shear disturbance in the interfacial transition zone, inhibiting the orderly growth of C-S-H around aggregates and causing uneven distribution of hydration products, forming high-density microcracks and localized weak layers. These defects severely impaired stress transfer and interfacial bonding performance, leading to significant reductions in splitting tensile strength and frost resistance. Importantly, such interfacial microcracks are often isolated, which, although markedly affecting mechanical and frost performance, rarely form continuous ion migration channels and thus have limited impact on the electrical flux test results.

Overall, timely and continuous vibration (as in group FQ) significantly enhanced chloride penetration resistance by increasing overall compaction, optimizing pore connectivity, and redistributing hydration products. Vibration applied during the initial setting period (group FB) showed some improvement in electric flux, but the accompanying interfacial damage reduced performance in freeze–thaw and abrasion resistance. Without vibration (group F), the higher pore connectivity resulted in the lowest resistance to chloride transport. These results highlight that vibration timing and duration play a decisive role in performance evolution. The improvement mechanism is mainly attributed to aggregate settlement and redistribution of hydration products, which generate a more complex pore network, increase tortuosity of transport paths, extend diffusion distances, and ultimately reduce effective chloride concentration while enhancing resistance to penetration [19,46].

#### 3.2.3. Abrasion Resistance

The mass loss of concrete under abrasion conditions is shown in Table 5. The results clearly indicate that the FB group experienced the greatest mass loss, corresponding to the poorest abrasion resistance, whereas the FQ and F groups exhibited similar mass loss values. This suggests that vibration applied from casting to final setting has a limited effect on abrasion resistance, while vibration applied from initial to final setting results in notable deterioration.

The deterioration mechanism is mainly attributed to microstructural disruption caused by delayed vibration. When vibration is applied after the concrete has entered the initial setting stage, the forming aggregate–paste skeleton is disturbed. This disruption leads to uneven redistribution of hydration products, weakening the protective function of the surface cement paste. Consequently, the bond strength between the aggregates and surrounding paste decreases, making the concrete surface more prone to spalling under abrasive loads and significantly reducing abrasion resistance.

Moreover, the high-slump concrete used in this study amplifies this effect. Due to its high matrix flowability, the concrete is more susceptible to segregation under external vibration, resulting in local aggregate enrichment or insufficient paste thickness. This non-uniformity creates preferential wear zones, accelerating surface paste loss and explaining the significant mass loss observed in the FB group. In contrast, vibration in the FQ group occurred while the paste was still in a fluid state, which could improve compaction, and thus its abrasion resistance was comparable to that of the non-vibrated control group (F group).

In summary, the effect of vibration on concrete abrasion resistance depends not only on the presence of vibration but also on the timing relative to hydration kinetics. Vibration applied from initial to final setting causes structural heterogeneity and weakens surface integrity, whereas vibration applied from casting to final setting enhances density and does not induce the same adverse effects.

## 4. Discussion

**Figure 21:**
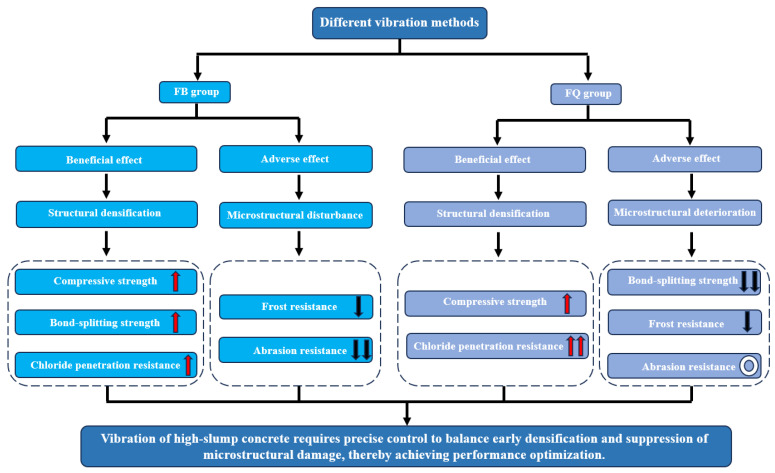
Schematic diagram of the effects of different vibration methods on the performance of high-slump concrete. Legend: An upward arrow to the right of the text indicates performance improvement, a downward arrow indicates performance degradation, and a circle denotes negligible performance change. The number of arrows represents the magnitude of the change.

Under different vibration methods, concrete shows complex and multi-dimensional performance changes. This is mainly due to vibration disturbing and reshaping its microstructure. Vibration has a dual effect: on one hand, proper vibration helps remove pores and improve density, which enhances early compressive strength and chloride penetration resistance; On the other hand, excessive or improper vibration can cause paste segregation and uneven distribution of hydration products, leading to microcracks in the interface zone, weaken bonding, and negatively affect bond-splitting strength, frost resistance, and abrasion resistance. The schematic diagram of the effects of different vibration methods on the performance of high-slump concrete is shown in Figure 21.

From the mechanical performance view, vibration significantly improved the early compressive strength of concrete. However, this advantage gradually diminished with curing time. At 28 days, the compressive strength of FQ and FB groups increased by only 16.1% and 16.2% compared to the control group F (Figure 9), indicating that the vibration effect is mainly reflected in the early stage. This trend is attributed to structural disturbances induced by vibration, especially microcracks and discontinuities in the interfacial transition zone, which hinder the uniform growth of hydration products and suppress later strength development. Existing studies also show that the effect of vibration on the compressive strength of concrete exhibits a certain degree of uncertainty. For example, Wang [47] reported that brief early-age disturbance can, to some extent, enhance the compressive strength of concrete. However, other researchers [22,48] have found that early-age disturbance may lead to a reduction in strength, which is often associated with excessively large vibration amplitudes or prolonged disturbance duration in the experiments. Bond-splitting strength exhibited greater sensitivity to vibration timing. At 28 days, the FB group showed a moderate enhancement with a disturbance coefficient of −5.8%, while the FQ group, subjected to vibration throughout the final setting stage, showed a significant reduction, with a disturbance coefficient as high as 32.8% (Figure 13), indicating severe weakening of the interfacial structure. SEM images confirmed these findings: in the FQ group, hydration products at the interface were sparsely distributed, the amount of C-S-H gel was low, and cracks were prevalent (Figure 15); by contrast, the F group exhibited a dense and uniform C-S-H network that effectively filled the interfacial cracks and enhanced bonding strength (Figure 16). Existing studies also indicate that the bond splitting strength is highly sensitive to external disturbances. Miguel Beltran et al. [49] reported that frequent traffic-induced vibrations can cause significant damage to the bonding interface between old and new concrete. Similarly, the findings of Sungnam Hong et al. [11] demonstrated that prolonged vibration duration ultimately reduces the bond strength between new and old concrete.

In terms of durability, performance indicators revealed both synergy and divergence. Vibration-induced microstructural reconstruction improved chloride penetration resistance, as evidenced by the FQ group achieving the lowest electric flux (Table 4). However, the formation of microcracks reduced freeze–thaw resistance and abrasion resistance. For instance, the FB group showed improved chloride resistance compared to F (1044 C vs. 1578 C), but its mass loss after 200 freeze–thaw cycles reached 2.8%, exceeding that of the F group (Figure 17). Moreover, the FB group exhibited the highest wear loss (Table 5), indicating the poorest abrasion resistance. These results suggest that vibration during the initial-to-final setting stage can severely disrupt the semi-solid structure. This “enhancement–degradation” duality was most evident in the FB group, highlighting the dominant role of vibration timing in performance evolution. Compared with mechanical properties, systematic research on the durability of disturbed concrete remains relatively limited. Some studies [50,51] have reported that early-age disturbance can induce micro-defects within the concrete, thereby weakening its frost resistance, which is consistent with the findings of this study. Wu et al. [52] observed that moderate vibration can improve the pore structure distribution of concrete and enhance its impermeability. However, other studies [23,53,54] have drawn opposite conclusions, suggesting that early-age disturbance may reduce the chloride penetration resistance of concrete. These discrepancies may be attributed to variations in concrete mix designs and vibration parameters across different studies. It is also noteworthy that abrasion resistance did not show a linear correlation with compressive strength. Although the FB group had moderate compressive strength (still higher than F at 28 days), it showed the worst abrasion resistance, suggesting that abrasion performance depends more on the integrity and uniformity of the paste–aggregate interface than on strength alone.

The microstructural analysis further revealed the underlying mechanisms of performance variation. SEM images showed that vibration significantly altered the formation and distribution of cement hydration products. In the FQ group, the interface displayed sparse C-S-H gel, reduced CH crystal size, isolated AFt, increased crack density, and enhanced pore connectivity (Figure 15). These microstructural defects are consistent with its lower bond-splitting strength at 28 days and higher freeze–thaw mass loss. In contrast, the F group exhibited a dense and continuous C-S-H network, with cracks effectively filled (Figure 16), and retained an intact surface and fewer cracks after freeze–thaw cycles (Figure 19a), reflecting superior bonding and durability. Although the FQ group demonstrated excellent resistance to chloride ion penetration, its microstructural defects limited its later bonding strength and freeze–thaw resistance. These findings confirm that vibration has a dual effect on concrete microstructure: it enhances compactness but disturb the interfacial structure. Therefore, careful control of vibration timing and duration is essential to optimize performance.

In summary, the influence of vibration on the performance of high-slump concrete is both time-dependent and dual in nature. Proper control of vibration timing and duration can improve early strength and compactness while minimizing microstructural damage, thereby ensuring long-term bonding and durability. In contrast, excessive or delayed vibration—especially during the initial-to-final setting stage—may cause irreversible damage, particularly at the fresh-to-old concrete interface and under freeze–thaw conditions. Thus, vibration parameters such as frequency, duration, and the concrete’s rheological state should be precisely managed in engineering practice to achieve optimal performance.

However, this study focused on laboratory-scale specimens and controlled vibration conditions, which may differ from field environments. The influence of vibration frequency, amplitude, and environmental factors such as temperature and humidity was not fully explored. Future studies should extend to large-scale tests and coupled simulations to further validate the findings.

## 5. Conclusions

This study investigates the performance evolution of high-slump concrete under different vibration timings. Through systematic testing of mechanical properties, durability, and microstructure, the main conclusions are as follows:

(1) Vibration effects on strength are highly time-dependent. Vibration significantly promotes early-age compressive strength, but the enhancement diminishes with curing age, indicating a clear time-sensitive effect.

(2) Bonding performance at the new–old concrete interface is extremely sensitive to vibration timing. Vibration applied from casting to final setting severely damages the interface, resulting in a marked reduction in bond-splitting strength. In contrast, vibration applied only during the initial to final setting period causes relatively minor interface damage and may even slightly improve strength.

(3) Different vibration modes produce a “deterioration–enhancement” pattern in durability. Vibration applied during the initial to final setting period tends to damage the forming microstructure, significantly reducing frost and abrasion resistance. Vibration applied from casting to final setting, while introducing minor micro-defects, improves pore structure and enhances chloride penetration resistance. This “performance differentiation” indicates that vibration timing affects various durability indicators in different directions.

(4) Microstructural observations reveal mechanisms behind performance evolution. SEM results show that vibration disperses hydration products, reduces C-S-H gel formation, and induces microcracks at the new–old interface, which are further exacerbated after freeze–thaw cycles.

## Figures and Tables

**Table 1:** Physical properties of cement.

Normal Consistency/%	Specific Surface Area (m^2^/kg)	Setting Time (min)		Compressive Strength (MPa)		Flexural Strength (MPa)	
		Initial	Final	3d	28d	3d	28d
24.7	345	115	195	24.3	56.6	4.8	8.6

**Table 2:** Mix proportions of concrete specimen.

Cement (kg/m^3^)	Water (kg/m^3^)	w/c	Sand (kg/m^3^)	Aggregate (kg/m^3^)		Polycarboxylate Superplasticizer (kg/m^3^)
				5–10 mm	10–20 mm	
520	156	0.3	674	330	770	3

**Table 3:** Test group design.

Test Group	Definition
F	Specimens in this group remained in a static environment from casting to final setting time
FB	Specimens in this group were subjected to vibration from the initial setting time to the final setting time
FQ	Specimens in this group were subjected to vibration continuously from casting until the final setting time

**Table 4:** The electric flux of concrete in chloride solution.

Group	Coulomb Electric Flux (C)	Resistance to Chloride Ion Permeability
F	1578	Low permeability
FB	1044	Low permeability
FQ	695	Very low permeability

**Table 5:** Mass loss of concrete under abrasion conditions.

Group	Abrasion Quality (kg/m^2^)
F	0.177
FB	0.311
FQ	0.177

## Data Availability

The original contributions presented in the study are included in the article. Further inquiries can be directed to the corresponding author.
